# The Impact of COVID-19 Related Distress on Antenatal Depression in Australia

**DOI:** 10.3390/ijerph20064783

**Published:** 2023-03-08

**Authors:** Lucy J. Frankham, Einar B. Thorsteinsson, Warren Bartik

**Affiliations:** Faculty of Medicine and Health, School of Psychology, University of New England, Armidale, NSW 2351, Australia

**Keywords:** antenatal depression, COVID-19, pregnancy, women, mental health, Edinburgh Postnatal Depression Scale

## Abstract

Globally, the impact of COVID-19 on mental health has been significant. Pregnant women are known to be a vulnerable population in relation to mental health. In Australia, there was an unprecedented demand during the pandemic for mental health services, including services for pregnant women. Maternal mental health has unique and enduring features that can significantly shape a child’s overall development and poor maternal mental health can have considerable social and economic costs. This cross-sectional study evaluated symptoms of antenatal depression and COVID-19-related distress in a sample of two hundred and sixty-nine pregnant women residing in Australia aged between 20 and 43 (*M* = 31.79, *SD* = 4.58), as part of a larger study. Social media advertising was used to recruit participants between September 2020 and November 2021. Prevalence rates for antenatal depression were found to be higher in this study (16.4%) compared with previous Australian prevalence rates (7%). COVID-19 distress in relation to having a baby during a COVID-19 outbreak significantly predicted symptoms of antenatal depression, *B* = 1.46, *p* < 0.001. Results from this study suggest that mothers and families may have increased mental health vulnerabilities as a consequence of the pandemic for some time yet.

## 1. Introduction

At the beginning of the COVID-19 pandemic, swift global action was required to mitigate the spread and impact of the virus. Australia initially responded by employing a suppression strategy using a number of targeted approaches including lockdowns [1]. Lockdowns were enforced across the country in early 2020, coinciding with Australia’s first COVID-19-related deaths. Lockdowns continued until the end of 2021 in various parts of the country. The suppression strategy and lockdowns were effective at eliminating the virus in the early stages of the pandemic but there was a substantial social and economic cost, including to the mental health and well-being of citizens [2,3]. Research on COVID-19 has demonstrated that women have been disproportionally impacted by the pandemic socially, emotionally, and economically [4,5,6]. These studies suggest that the pandemic resulted in an increased burden on women, who were often expected to take on additional responsibilities such as caregiving, household management, and working from home, while also experiencing disruptions in their day-to-day living, employment, and financial security and that the increased demands on women’s resources may have led to greater stress and decreased well-being. Rates of family violence towards women also increased during lockdowns [5]. Data from these studies show that the indirect impact of COVID-19 on women is complex and multi-factorial.

The increased emotional distress associated with the additional responsibilities and risks placed on women during the pandemic may be understood using the Power Threat Meaning Framework (PTMF) developed by the British Psychological Society [7]. The Power Threat Meaning Framework offers a holistic and empowering approach to understanding and addressing the impact of power imbalances caused by social, cultural, and political factors. PTMF moves away from traditional medical and psychiatric models, instead understanding mental distress through the lens of what has happened to an individual (power), how it affects them (threat), what this means to them (meaning), and how they survived or coped (threat response) [7,8]. The model has been used both to conceptualize and understand distress in pregnancy [8] and in relation to COVID-19 [9]), as well as a host of other circumstances where mental and emotional distress arises. Ordinarily, women are up to twice as likely to experience depression than men and are more vulnerable during childbearing years [10,11,12], which can also be explained using the PTMF. During the pandemic, the mental health of young women has been among the most impacted [13] with the focus predominantly on mitigation efforts and disease control, less attention was given to the impact of the pandemic on mental health.

From a maternal mental health perspective, there is increasing evidence that pregnant women have been significantly impacted by the pandemic. Consistent with the perspectives of the Power Threat Meaning Framework, studies have consistently found antenatal depression rates to be higher during the pandemic than pre-pandemic rates across the globe [14,15,16,17,18,19]. Higher antenatal depression scores have also been found to be associated with disease mitigation measures and COVID-19 related distress across studies [15,16,18,19,20]. In many instances, mitigation efforts were at odds with usual best practice. The way routine care was delivered changed, many appointments were modified (e.g., changed to telehealth or less regular), visitors and support people were minimized or restricted altogether, and antenatal education cancelled [16,21,22,23]. Such measures served to decrease support for mothers at a time when it was most needed. Support is known to be a vital buffer for perinatal mental health issues, together with the standard of maternity care available to women, they have a valuable influence on the well-being of mothers and infants [24,25,26,27]. The nature and standard of maternity care provided can significantly reduce rates of infant mortality and medical intervention [28]. Optimally women should be able to access a high standard of conventional healthcare that includes comprehensive screening and informal supports including other community health interventions [26]. The mitigation efforts of the pandemic (e.g., lockdowns, reduced appointments, and visitor restrictions) likely compromised the usual systems and supports available to pregnant women in Australia.

Maternal mental health concerns possess unique and potentially enduring features that make them different from other mental health concerns, a consideration that is often misunderstood or overlooked. The perinatal period (typically described as between pregnancy and one year postnatal) is known to be associated with increased vulnerability to mental health concerns [29,30]. Beginning from pregnancy, the impact of antenatal depression has even been described as a developmental cascade to future mental health problems for both mothers and their children [31]. This begins with an increased likelihood of adverse perinatal outcomes for infants of mothers with antenatal depression. These infants are more likely to be born earlier, have lower birth weight, and are less likely to be breastfed exclusively at birth [32]. Mothers who experience depression during pregnancy are also more likely to experience it again during their child’s lifetime and are more likely to go on to develop postnatal depression [27,31,32]. Children whose mothers have had postnatal depression are more likely to have problems with physical health such as asthma and respiratory problems, impaired immune system responses, and neurodevelopmental issues as well as behavioural and emotional concerns such as attention deficit hyperactivity disorder and others [26,33,34]. Postnatal depression can impact the quality of mother-infant relationships [35,36], which can lead to an increased risk of aggression, emotional difficulties, academic problems, and poor self-worth [37]. Due to this enduring impact of poor maternal mental health on infants and children, maternal mental health concerns are a major social and economic issue that should not be underestimated. The long-term economic cost of perinatal depression on the community is considerable and the burden on the child is significant [24,25,26,28,33,38,39,40,41,42]. For example, one UK study found nearly three-quarters (72%) of the total public health cost relates to adverse impacts on the child, rather than the mother [39]. One way of reducing the social and economic cost is to support women better during pregnancy.

The aim of the current study was to examine the impact of COVID-19-related distress on depression in pregnant women. Understanding the impact of the pandemic on pregnant women may help to inform practices and policies to better support the currently affected cohort in the short term and also help to inform responses and policies relating to any future health crises.

## 2. Materials and Methods

### 2.1. Participants and Sample Characteristics

Paid social media advertising was used to recruit 269 participants (*M* = 31.79, *SD* = 4.58, from 23–40 years of age), between September 2020 and November 2021, as part of a larger study with sample size determined by the requirements of the larger study. The sample mostly comprised Caucasian nulliparous women with a university education planning a hospital birth in urban Australia. Four reported being in a same-sex relationship and nine participants were not in a relationship, see Table 1 for further sample characteristics.

Participants were asked to complete an online survey and were offered entry in a draw for a gift voucher as an incentive to participate. After reading the information statement at the beginning of the survey, participants were required to click a proceed button in order to continue, indicating consent. Information and contact details for mental health support services were presented to each participant twice within the survey. The inclusion requirements for participants were: (a) 18 years old or over, (b) greater than 12 weeks pregnant, (c) English speaking, and (d) living in Australia. The present project was approved by the relevant Human Research Ethics Committee.

### 2.2. Measures

#### 2.2.1. Edinburgh Postnatal Depression Scale

The Edinburgh Postnatal Depression Scale (EDPS) was used to measure symptoms of antenatal depression [43]. The EPDS is a 10-item self-report questionnaire with a maximum score of 30. Higher scores on the scale indicate higher levels of distress. The EPDS has good reported validity for assessing perinatal distress [44,45,46,47,48]. The EPDS has good reliability and validity [47,48] including high test-retest reliability in pregnancy [α = 0.82–0.84; 45]. The EDPS has also been found to be reliable with women from culturally and linguistically diverse backgrounds [49]. A cut-off score of ≥13 on the EPDS for probable depression was employed to calculate the prevalence rate found in this study, consistent with other Australian studies [43,50] and other studies focused on COVID-19 [14]. Pre-pandemic rates of antenatal depression in Australia measured with the EPDS are reported to be around 7% [32,40,51].

#### 2.2.2. COVID-19 Distress

At this time of the study development, there were no reliable and valid measures of COVID-19 distress. Instead, COVID-19-related distress was measured using a simple two-item five-point Likert scale rated from 1 (no concern) to 5 (extremely concerned). Participants were asked “In relation to having your baby, how concerned are you as a result of the COVID-19 outbreak?” and “Overall, how concerned are you as a result of the COVID-19 outbreak?”. The two items were found to be correlated with each other (*r* = 0.72) indicating reliability for the COVID-19 distress measure.

#### 2.2.3. Data Analysis

The Statistical Package for the Social Sciences 27 (SPSS.27) program (IBM corp., New York, NY, USA) was used for analyses. The present study was a cross-sectional correlational design. Data were checked for accuracy, there were no outliers or missing data.

Scores on the Edinburgh Postnatal Depression Scale were low overall and found to be significantly skewed (*z_skew_* > 3.29). EDPS scores in a non-clinical sample are expected to be positively skewed, therefore this sample is representative of a non-clinical population [32,43]. In order to address the skewed data, it was decided the most appropriate action for the non-normally distributed variables was to transform them (using the SPSS SQRT function), as recommended by Tabachnick and Fidell (2018) for moderately skewed variables. The transformed data did not alter the substantive interpretation of the data, as such the untransformed data was retained in order to make it easier to relate back to the original data [52].

A single linear regression analysis testing whether COVID-19-related distress predicted higher depression scores was conducted. The COVID-19 distress overall variable was not included in the regression analysis due to multicollinearity. None of the participant demographic characteristics were included as there was insufficient distribution across categories.

## 3. Results

### 3.1. Descriptive Statistics

#### 3.1.1. Prevalence of Antenatal Depression

The cut-off for probable depression suggested the prevalence rate for antenatal depression in this sample (*N* = 269) was 16.4% compared with Australia’s pre-pandemic rate of around 7% [32,40,51].

#### 3.1.2. COVID-19 Related Distress

Overall distress scores indicated the average mother to be ‘a little to moderately concerned’ (*M* = 2.60, *SD* = 1.00) about the impact of COVID-19 on them.

Scores for COVID-19 Distress in relation to having their baby indicated that on average mothers were ‘a little to moderately concerned’ (2.42, *SD* = 1.01) about the impact of COVID-19 on their pregnancy and birth.

### 3.2. Main Analyses

There was a positive association between COVID-19-related distress overall *r*(267) = 0.17, *p* = 0.005 and there was also a positive association in relation to having a baby during a COVID-19 outbreak *r*(267) = 0.27, *p* < 0.001. Age was not significantly related to either of the key variables (antenatal depression symptoms *r*(267) < 0.01, *p* > 0.05; COVID-19 in relation to having a baby *r*(267) = 0.01, *p* > 0.05. Associations between other categorical characteristics (e.g., location) were not tested due to insufficient variation across categories.

As can be seen in Table 2, the overall regression model was significant, *R²* = 0.07, *F*(1, 267) = 20.43, *p* < 0.001. It was found that COVID-19 distress in relation to having a baby significantly predicted symptoms of antenatal depression, *B* = 1.46, *p* < 0.001. Indicating that distress about COVID-19 in relation to a woman’s baby was an important factor for the experience of antenatal depression during the pandemic. Given that only 7% of the variance is explained by the model tested, it is probable there may be other contributing factors that were not measured in this study.

## 4. Discussion

The current study adds to the small body of Australian research showing that, consistent with the rest of the globe, during the COVID-19 pandemic women in Australia were experiencing elevated depressive symptoms during pregnancy [15,16]. Women in the present study were on average moderately concerned about COVID-19 overall and in relation to their baby during the pandemic. Both COVID-19 distress variables were associated with more symptoms of antenatal depression. Prevalence rates of probable antenatal depression (EPDS scores ≥ 13) in this sample were more than twice Australia’s pre-pandemic rates, indicating that overall, the women in this study were coping poorer than usual. The findings suggest that the uncertainty and anxiety around the impact of the pandemic on pregnancy and childbirth may have been a significant stressor for pregnant women. Further, COVID-19 distress in relation to having a baby significantly predicted symptoms of antenatal depression. While the predictive power of the model overall was low, the findings demonstrate that COVID-19 distress in relation to having a baby was important and, as indicated in other studies, there is likely a number of other variables that were influencing stress and depressive symptoms. Studies from a number of other countries have found a range of factors to be associated with the observed increase in rates of antenatal depression during the pandemic including: COVID-19 mitigation efforts (e.g., changes to pregnancy care, social distancing), COVID-19-related distress (e.g., exposure to media, COVID-19 case numbers) and existing risk or vulnerability factors (e.g., previous depression, poor social support) [15,16,18,19,20]. Specifically in Australia, another larger study found existing psychosocial influences such as family stress and lower social support, as well as the mitigation response (e.g., social distancing and changes to maternity care), predicted greater antenatal depression symptoms [15], indicating that aspects of both maternity care and informal supports were involved.

Findings from this study and others suggest that aspects of both the pandemic and the pandemic response evaporated the capacity of these formal and informal systems to provide quality care, subsequently negatively impacting maternal mental health. During its peak, the pandemic disrupted healthcare systems and social support networks, as well as created economic and employment uncertainties, which seems to have contributed to increased stress and mental health concerns for pregnant women. The impact of these influencing factors is consistent with the perspectives of the Power Threat Meaning Framework, which asserts that mental distress can be explained by social, economic, and political adversities [7]. Since women were disproportionately impacted by the pandemic in so many ways [4,5,6,53], it makes sense that many women were finding it difficult to cope and experiencing symptoms of depression, especially during pregnancy. Part of this may be because there are more systemic issues at play impacting women, which umbrella above the individual psychosocial and demographical characteristics of women. This would suggest that the impact of the pandemic on women’s mental health is influenced not only by individual characteristics and experiences, but also by more systemic issues such as the influences of patriarchy and capitalism that are known to negatively impact women [53,54,55,56,57].

Comprehensive maternity care that is of a high standard combined with informal supports is known to be an important protective influence on maternal mental health that can help to buffer existing vulnerabilities and the more immediate stressors of pregnancy and motherhood [25,26,28,58]. Poor pregnancy support is linked to greater adverse infant outcomes and poorer maternal mental health [26,33,59]. Maternity care is ultimately one of the most early and important points of intervention for the population’s physical and mental health across the lifespan. Studies in other extreme examples such as those following children of mothers that were pregnant during natural disasters have shown disturbingly high levels of internalizing and externalizing problems such as ADHD, depression, and anxiety, as well as poorer language and cognitive development in the children that were exposed to natural disasters in-utero [59,60,61]. Environmental stress is known to impact the long-term health of a developing fetus through a number of mechanisms including epigenetics (changes to developmental programming) and shortening of telomeres (an important protective component at the end of chromosomes) [58]. In these various ways, right down to the cellular level, environmental stress in-utero can have long-term health effects by changing the programming of organs, tissues, and body system structures. These changes can lead to an increased risk of metabolic, cardiovascular, immunological, neurobehavioral problems, and cancer [58]. While we have known much of this for a long time, we continue to minimize the importance of psychosocial and environmental factors in pregnancy and as observed during the pandemic, tend to treat pregnancy and birth more as a medical event. Addressing symptoms of antenatal depression and other mental health concerns during a pandemic or other major stressful events perhaps requires a more holistic approach that takes into account both individual and systemic factors in order to ameliorate the impact of this stress on women. Further research may involve exploring ways to increase screening of pregnant women and more targeted psychosocial interventions, as well as broader efforts to address the oppression and socioeconomic inequality women face ordinarily, which was exacerbated during the pandemic [5,6,57].

### Limitations, Implications, and Future Research

This study was a cross-sectional design with a small sample size drawn from a convenience sample recruited on social media. The majority of participants were Caucasian university-educated partnered women from an urban location. The study sample was fairly homogenous and therefore had insufficient power to explore potential influences of participant characteristics such as education or location. Factors such as existing risk factors and more specific COVID-19-related factors were not measured. It is also not possible to delineate exactly what aspects of COVID-19 distress in relation to having a baby contributed to the increased antenatal depression scores in the current sample. This was reflected in the low predictive power of the regression model, suggesting that there were other important influences that were not measured in this study. Moreover, even though antenatal depression prevalence rates in Australia were found to be higher in this sample, overall, this finding was much lower than increases observed in other countries [14,17,19,20]. Another Australian study [15] also observed rates much higher (26.5%) than the present study (16.4%). This difference in the Australian studies may reflect the smaller sample size in the present study but also the timing of the data collection may be a factor with the present study data collected over a longer period (September 2020–November 2021) compared with August 2020 to February 2021 in Lequertier et al.’s study. Australia’s vaccine rollout began in February 2021 [62] perhaps this contributed to reducing distress among some women, which was then reflected in the present study. Another difference between the two Australian studies is the rate of public healthcare consumption. In the present study, more than 90% of women were accessing public healthcare, while in the study by Lequertier et al. [15], only 40.8%. It may be that there was something different that was experienced in public healthcare or that more distressed women chose not to utilise public healthcare. Regardless of these differences, the study by Lequertier et al. [15] supports the findings of the present study, indicating that COVID-19-related distress in Australian women was associated with increased symptoms of antenatal depression during 2020 and 2021. Future research may want to explore and unpick what factors contributed most to the COVID-19-related distress, this would help to plan better for any future crises and potentially reduce the impact on maternal mental health.

## 5. Conclusions

The COVID-19 pandemic was an unprecedented global emergency for modern times. Understanding the impact of the pandemic on maternal mental health is important and valuable in understanding ways to manage any future crises that may impact the treatment of pregnant women. This study and others have consistently found during the pandemic women experienced high levels of distress and these increases in distress were related to having more depressive symptoms during pregnancy. Recommendations from other studies suggest that increasing the screening of pregnant women and ramping up of supports may help mitigate the impact of long-term negative outcomes [14,17,20], this recommendation will be important for any future global crisis. Additionally, reviewing maternity care and support provisions using the Power Threat Meaning Framework may also help to scrutinize future disease management strategies in a less psychologically detrimental way. The framework would allow social, economic, and political factors to be considered in any future responses to maternity care during a pandemic, in addition to medical care. Extending access to programs such as sustained nurse home visiting, that support families with health, education, and early parenting beginning in pregnancy may be one way to combat some of these issues. Evidence for nurse home visiting programs is substantial [63,64]. Studies of such programs show families who participate report considerable parenting, social, and health benefits as much as five years later, indicating the programs can provide valuable lifelong improvement to the well-being of families.

In terms of the current health crisis, ongoing additional screening and support for women and their children may be important for those families that were pregnant during the peak of the crisis in 2020 and 2021 to minimize any cascade effect related to the increase of antenatal depression symptoms observed in this study and many others, which may in turn have lifelong consequences for the child. Maintaining vigilance with screening and support for this cohort would potentially reduce future social and economic costs.

## Figures and Tables

**Table 1:** Sample Characteristics (*N* = 269).

Characteristic	*n*	%
Nulliparous	208	77.3
Multiparous	61	22.7
**Ethnic Background**		
Aboriginal or Torres Strait Islander	2	0.7
White European	186	69.1
Indian	8	3.0
Asian	28	10.4
Middle Eastern	4	1.5
North American	2	0.7
South American	2	0.7
Mixed race	16	5.9
Other	20	7.4
Prefer not to say	1	0.4
**Geographical location**		
Urban/City	192	71.4
Rural	69	25.7
Remote	8	3.0
**In a relationship**		
Yes	260	96.7
No	9	3.3
**Same sex relationship**		
Yes	4	1.5
No	264	98.1
Prefer not to say	1	0.4
**Education**		
No formal qualifications	7	2.6
Completed high school	22	8.2
TAFE certificate/diploma	65	24.2
University degree	175	65.1
**Birth**		
Single births	266	98.9
Multiple birth	3	1.1
Birth education classes—yes	157	58.4
Birth education classes—no	112	41.6
Birthing in hospital	244	90.7
Birthing in private birth centre	7	2.6
Birthing in home environment	18	6.7

**Table 2:** Linear Regression Analysis Predicting Antenatal Depression Symptoms from COVID-19 Related Distress in relation to having a baby and overall (*N* = 269).

Predictor	β	*B*	*SE B*	95% CI for *B*
COVID-19 Distress Baby	0.27	1.46 *	0.32	[0.83, 2.10]

* *p* < 0.001.

## Data Availability

The data presented in this study are openly available in FigShare at 10.6084/m9.figshare.21708551.
